# Donor Human Milk Protects against Bronchopulmonary Dysplasia: A Systematic Review and Meta-Analysis

**DOI:** 10.3390/nu10020238

**Published:** 2018-02-20

**Authors:** Eduardo Villamor-Martínez, Maria Pierro, Giacomo Cavallaro, Fabio Mosca, Boris W. Kramer, Eduardo Villamor

**Affiliations:** 1 Department of Pediatrics, Maastricht University Medical Center (MUMC+), School for Oncology and Developmental Biology (GROW), 6202 AZ Maastricht, The Netherlands; evillamorm@gmail.com (E.V.-M.); b.kramer@mumc.nl (B.W.K.); 2 Neonatal Intensive Care Unit, Alessandro Manzoni Hospital, 23900 Lecco, Italy; maria.pierro93@gmail.com; 3 Neonatal Intensive Care Unit, Department of Clinical Sciences and Community Health, Fondazione IRCCS Cà Granda Ospedale Maggiore Policlinico, Università degli Studi di Milano, 20122 Milan, Italy; giacomo.cavallaro@mangiagalli.it (G.C.); fabio.mosca@mangiagalli.it (F.M.)

**Keywords:** donor human milk, bronchopulmonary dysplasia, breast milk, preterm formula, pasteurization

## Abstract

Bronchopulmonary dysplasia (BPD) is the most common complication after preterm birth. Pasteurized donor human milk (DHM) has increasingly become the standard of care for very preterm infants over the use of preterm formula (PF) if the mother’s own milk (MOM) is unavailable. Studies have reported beneficial effects of DHM on BPD. We conducted a systematic review and meta-analysis of randomized controlled trials (RCTs) and observational studies on the effects of DHM on BPD and other respiratory outcomes. Eighteen studies met the inclusion criteria. Meta-analysis of RCTs could not demonstrate that supplementation of MOM with DHM reduced BPD when compared to PF (three studies, risk ratio (RR) 0.89, 95% confidence interval (CI) 0.60–1.32). However, meta-analysis of observational studies showed that DHM supplementation reduced BPD (8 studies, RR 0.78, 95% CI 0.67–0.90). An exclusive human milk diet reduced the risk of BPD, compared to a diet with PF and/or bovine milk-based fortifier (three studies, RR 0.80, 95% CI 0.68–0.95). Feeding raw MOM, compared to feeding pasteurized MOM, protected against BPD (two studies, RR 0.77, 95% CI 0.62–0.96). In conclusion, our data suggest that DHM protects against BPD in very preterm infants.

## 1. Introduction

The nutritional and immunological benefits of providing human milk to very preterm (gestational age (GA) < 32 weeks) or very low birth weight (VLBW, i.e., birth weight < 1500 g) infants have been increasingly recognized [1,2,3,4,5]. Official bodies such as the World Health Organisation [6], American Academy of Paediatrics [7], or the European Society for Paediatric Gastroenterology, Hepatology, and Nutrition [8] recommend mother’s own milk (MOM) as the first choice in VLBW infant feeding and advocate for making strong efforts to promote lactation. When MOM is not available, pasteurized donor human milk (DHM) is the preferred choice [7,8]. When MOM and DHM are not available, preterm formula (PF) should be used [8]. 

Although evidence exists regarding the protective effects of MOM in reducing the risk of necrotizing enterocolitis (NEC), late-onset sepsis (LOS), and retinopathy of prematurity (ROP) in VLBW infants, questions remain regarding whether DHM provides the same benefits [5,9,10,11,12,13,14,15]. DHM is usually donated by mothers of term infants and is pasteurized. Therefore, numerous MOM components which could contribute to protect against adverse outcomes of prematurity are reduced or absent in DHM [15,16,17].

Bronchopulmonary dysplasia (BPD) is the most common complication of extreme preterm birth. Infants who develop BPD manifest aberrant or arrested pulmonary development and can experience lifelong alterations in cardiopulmonary function [18,19]. Optimal nutritional support is considered a cornerstone in the treatment/prevention of BPD [18,20]. Several studies have reported protective effects of DHM in the development of BPD [10,21]. However, a systematic review of the evidence in the literature has not been performed to date. Therefore, we aimed to conduct a systematic review and meta-analysis of the interventional and observational studies reporting data on the effects of pasteurized DHM on BPD as well as other indicators of pulmonary outcome in preterm infants. 

## 2. Materials and Methods

A protocol was developed prospectively that detailed the specific objectives, criteria for study selection, the approach to assessing study quality, clinical outcomes, and statistical methodology. The study was carried out and reported according to the guidelines of the Preferred Reporting Items for Systematic Reviews and Meta-Analysis (PRISMA) [22]. The PRISMA checklist for this report can be found in supplementary material (Table S1).

### 2.1. Data Sources and Search Strategies

A comprehensive literature search was conducted using PubMed/MEDLINE and EMBASE, from their inception to 1 December 2017. The search strategy for PubMed used the following terms, including Mesh terms: (breast milk OR infant feeding OR donor milk OR pasteurized human milk OR preterm formula) AND (preterm infant OR very low birth weight infant) AND (outcome OR bronchopulmonary dysplasia OR BPD). A similar strategy was used in the other databases. No language limit was applied. Translation was performed where necessary. Randomized controlled trials (RCTs) and observational studies were included in the review. Narrative reviews, systematic reviews, case reports, letters, editorials, and commentaries were excluded, but read to identify potential additional studies. Additional strategies to identify studies included use of “related articles” feature on PubMed, and use of “cited by” tool in Web of Science and Google Scholar.

### 2.2. Eligibility Criteria and Study Selection

Studies were included if they were RCTs, cohort studies or case-control studies, involving the use of DHM in very preterm (GA < 32 weeks) or VLBW (BW < 1500 g) infants, included a study and control group divided according to feeding policy, and reported results on BPD, days of mechanical ventilation (MV) or days on oxygen (O_2_). BPD was defined as oxygen dependency at 28 days (BPD28) or as oxygen dependency at 36 weeks post-menstrual age (BPD36). To identify relevant studies, two reviewers (E.V.-M., E.V.) independently screened the results of the searches and applied inclusion criteria using a structured form. Discrepancies were identified and resolved through discussion or in consultation with the other researchers.

### 2.3. Data Extraction and Assessment of Risk of Bias

Two investigators (E.V.-M., E.V.) extracted the data by using a data collection form designed for this review. The following information was collected: study type, number of patients, number and name of centres, study period, inclusion/exclusion criteria, patient characteristics (GA, BW), feeding intervention or observation (MOM, DHM, and/or PF, type of fortifier, duration of intervention/observation), and outcome (incidence of BPD, days on mechanical ventilation, days on oxygen). Two other investigators (M.P., G.C.) independently validated the accuracy of the extracted data. 

Two reviewers (E.V.-M., E.V.) independently assessed risk of bias in each study, using two predetermined tools. Risk of bias in RCTs was assessed by using the Cochrane “Risk of Bias Assessment Tool” [23]. For each domain (random number generation, allocation concealment, blinding of intervention and outcome assessors, completeness of follow-up, selectivity of reporting, and other potential sources of bias) the risk was assessed as low, high, or unclear. Risk of bias in observational studies was assessed using the Newcastle-Ottawa scale for quality assessment of cohort and case-control studies [24]. This scale uses a rating system (range: 0–9) that gives points for selection (0–4), comparability (0–2), and outcome/exposure (0–3). Discrepancies during the data extraction and assessment of the risk of bias process were resolved by discussion and consensus among all reviewers.

### 2.4. Statistical Analysis

Studies were combined and analysed using comprehensive meta-analysis V 3.0 software (Biostat Inc., Englewood, NJ, USA). For dichotomous outcomes, the Mantel-Haenszel (MH) risk ratio (RR) with a 95% confidence interval (CI) was calculated from the data provided in the studies. For continuous outcomes, the mean difference (MD) with 95% CI was calculated. When studies reported continuous variables as median and range or interquartile range, we asked authors for mean and standard deviation (SD). If they did not provide the requested data, we estimated the mean and SD using the method of Wan et al. [25]. 

Due to anticipated heterogeneity, summary statistics were calculated with a random-effects model. This model accounts for variability between studies as well as within studies. Subgroup analyses were conducted according to the mixed-effects model [26]. In this model, a random-effects model is used to combine studies within each subgroup and a fixed-effect model is used to combine subgroups and yield the overall effect. The study-to-study variance (tau-squared) is not assumed to be the same for all subgroups. This value is computed within subgroups and not pooled across subgroups. Statistical heterogeneity was assessed by Cochran’s *Q* statistic and by the *I*^2^ statistic, which is derived from *Q* and describes the proportion of total variation that is due to heterogeneity beyond chance [27]. We used the Egger’s regression test and funnel plots to assess publication bias. A probability value of less than 0.05 (0.10 for heterogeneity) was considered statistically significant.

## 3. Results

**Figure 1:**
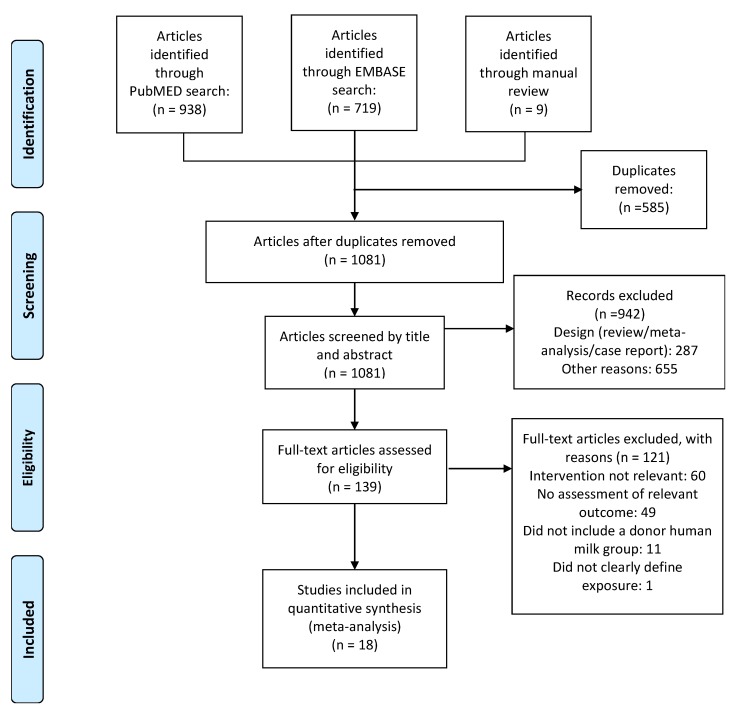
Flow diagram of the literature search process.

Based on the titles and abstracts of 1081 citations, we identified 139 potentially relevant studies (Figure 1), 18 of which met the inclusion criteria [10,21,28,29,30,31,32,33,34,35,36,37,38,39,40,41,42,43]. Seven studies [10,30,31,38,40,41,43] were RCTs and 11 studies [21,28,29,32,33,34,35,36,37,39,42] were observational. The main characteristics of the included RCTs are shown in Table 1, and the main characteristics of the included observational studies are shown in Table 2. Definitions of feeding type varied across studies. None of the studies had BPD as their primary outcome. To pool data, we classified feeding type comparisons into four categories: (1) DHM vs. PF; (2) MOM vs. DHM; (3) raw MOM vs. pasteurized MOM; and (4) raw MOM vs. frozen MOM.

### 3.1. Quality Assessment and Publication Bias

Six of the seven RCTs [10,30,31,40,41,43] included in the review scored low risk of bias for random sequence generation, allocation concealment, incomplete outcome data, selective reporting and other bias (Table A1). Four studies [10,31,38,40] scored high or unknown risk of bias on blinding (Table A1). The 12 observational studies included scored between 6 and 9 points on the Newcastle-Ottawa scale, out of a possible 9 points (Table A2). The median score of included studies was 7 points. 

We found no evidence of publication bias in any of the analyses that we performed, either through visual inspection of funnel plots or Egger’s regression intercept. However, the limited amount of studies per outcome makes these tests inconclusive.

### 3.2. Randomized Controlled Trials: Donor Human Milk vs. Preterm Formula

**Figure 2:**
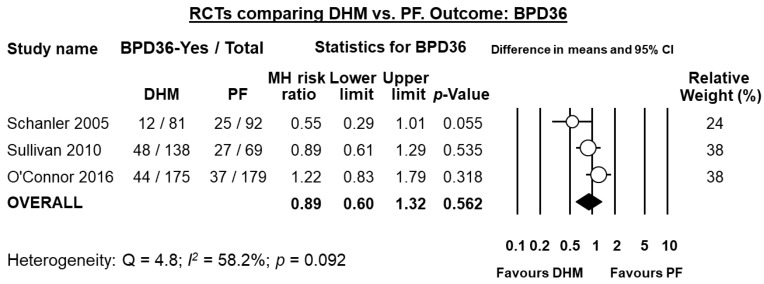
Meta-analysis of randomized controlled trials assessing the effects of supplementation of MOM with DHM, compared with supplementation with PF, on risk of BPD36. Circles (O) represent the effect sizes per study, and their size represents the relative weight of the study in the meta-analysis. Diamonds (◆) represent the pooled effect size. DHM: donor human milk; PF: preterm formula; BPD36: bronchopulmonary dysplasia defined as oxygen dependency at 36 weeks post-menstrual age; MH: Mantel-Haenszel.

Five RCTs [10,30,40,41,43] randomized infants to receive DHM or PF when MOM was insufficiently available. Thus, they compared infants receiving MOM supplemented with DHM vs. infants receiving MOM supplemented with PF. Three of these studies [10,40,41] reported on the rate of BPD36. As shown in Figure 2, meta-analysis could not detect a statistically significant effect of DHM on BPD36 (RR 0.89, 95% CI 0.60 to 1.32, *p* = 0.562). The RCT of Sullivan et al. used a DHM-based fortifier in the DHM group, and a bovine milk-based fortifier in the PF group. The studies of Schanler et al. [10] and O’Connor et al. [41] used a bovine milk-based fortifier in both groups. Exclusion of the study of Sullivan et al. [40] for using a different type of fortifier did not significantly affect the results of the meta-analysis on BPD36 (RR 0.85, 95% CI 0.39 to 1.85, *p* = 0.676). One study [30] reported on BPD28 and could not find any significant effect (RR 1.06, 95% CI 0.75 to 1.51, *p* = 0.724). 

**Figure 3:**
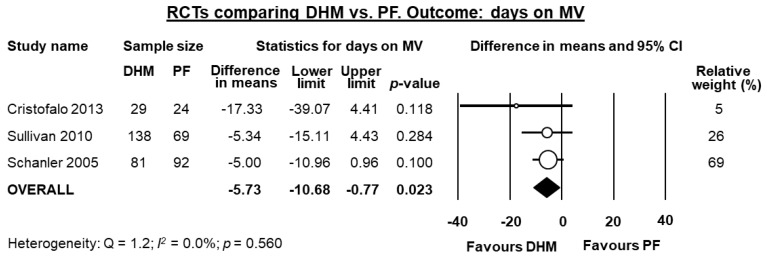
Meta-analysis of randomized controlled trials assessing the effects of supplementation of MOM with DHM, compared with supplementation with PF, on mean days on MV. Circles (O) represent the effect sizes per study, and their size represents the relative weight of the study in the meta-analysis. Diamonds (◆) represent the pooled effect size. DHM: donor human milk; PF: preterm formula; MV: mechanical ventilation.

Three studies [10,40,43] reported data on days on mechanical ventilation, and meta-analysis showed a significant reduction in this outcome for infants receiving DHM (MD −5.73 days, 95% CI −10.68 to −0.77, *p* = 0.023, Figure 3). Two studies [40,43] reported data on days on oxygen, and meta-analysis could not find a significant reduction in this outcome for infants receiving DHM (MD −9.11 days, 95% CI −24.82 to 6.60, *p* = 0.256).

### 3.3. Observational Studies: Donor Human Milk vs. Preterm Formula

**Figure 4:**
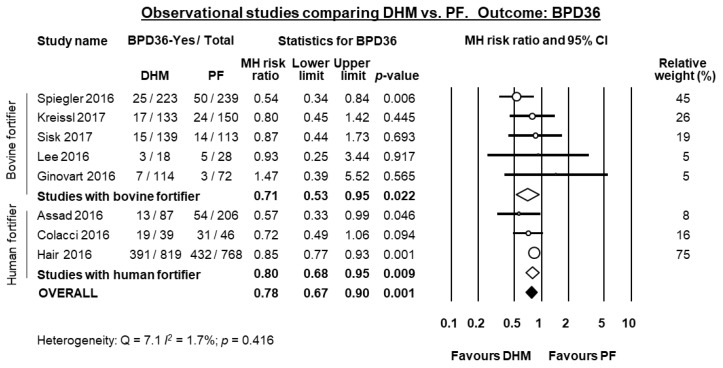
Meta-analysis of observational studies assessing the effects of supplementation of MOM with DHM, compared with supplementation with PF, on risk of BPD36. Circles (O) represent the effect sizes per study, and their size represents the relative weight of the study in the meta-analysis. Diamonds (◆) represent the pooled effect size. DHM: donor human milk; PF: preterm formula; BPD36: bronchopulmonary dysplasia defined as oxygen depenency at 36 weeks post-menstrual age; MH: Mantel-Haenszel.

Eight observational studies compared infants receiving MOM supplemented with DHM to infants receiving MOM supplemented with PF [21,28,29,33,35,36,37,39] of which all reported data on BPD36. Three of these studies [28,29,35] used a DHM-based fortifier in the DHM-group, and a bovine milk-based fortifier in the PF group. When these three studies were pooled, meta-analysis showed a significant protective effect of DHM on BPD36 (RR 0.80, 95% CI 0.68 to 0.95, *p* = 0.009, Figure 4). This protective effect of DHM on BPD36 was also observed when the five studies [21,33,36,37,39] that did not use DHM-based fortifier were pooled (RR 0.71, 95% CI 0.53 to 0.95, *p* = 0.022, Figure 4) and when the 8 studies were combined in a mixed-effects meta-analysis (RR 0.78, 95% CI 0.67 to 0.89, *p* = 0.0005, Figure 4). The study of Ginovart et al. [33] also reported data on BPD28 and found no significant difference in this outcome between the group receiving DHM and the group receiving PF (RR 1.18, 95% CI 0.68 to 2.05, *p* = 0.560). 

**Figure 5:**
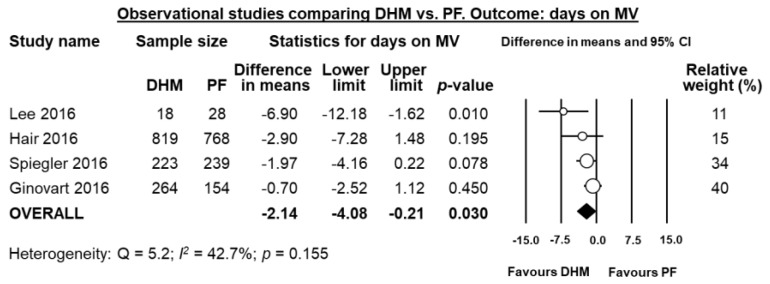
Meta-analysis of observational studies assessing the effects of supplementation of MOM with DHM, compared with supplementation with PF, on mean days on mechanical ventilation. Circles (O) represent the effect sizes per study, and their size represents the relative weight of the study in the meta-analysis. Diamonds (◆) represent the pooled effect size. DHM: donor human milk; PF: preterm formula.

**Figure 6:**
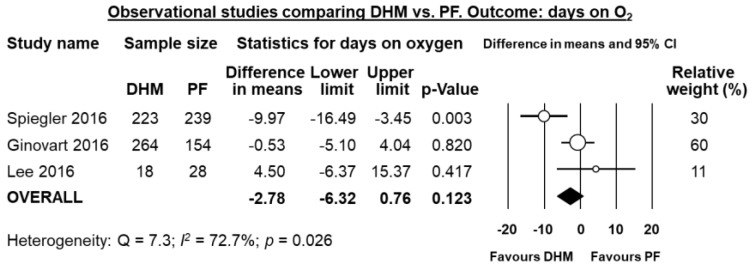
Meta-analysis of observational studies assessing the effects of supplementation of MOM with DHM, compared with supplementation with PF, on mean days on oxygen. Circles (O) represent the effect sizes per study, and their size represents the relative weight of the study in the meta-analysis. Diamonds (◆) represent the pooled effect size. DHM: donor human milk; PF: preterm formula.

Four studies [21,33,35,37] reported data on mean days on mechanical ventilation, and meta-analysis showed a significant reduction in difference in means in the DHM-group (MD 2.14 days, 95% CI −4.08 to −0.21, *p* = 0.030, Figure 5). Three studies [21,33,37] reported data mean on days on oxygen, and meta-analysis could not find a significant difference in mean days on oxygen in the DHM group (MD −2.78 days, 95% CI −6.32 to 0.76, *p* = 0.123, Figure 6).

### 3.4. Mother’s Own Milk vs. Donor Human Milk

Five observational studies [10,28,34,39,42] compared infants who received mainly MOM vs. infants who received mainly DHM. There were significant differences in group design. Giuliani et al. [34] compared infants receiving >80% MOM (supplemented with <20% DHM) vs. infants receiving <20% MOM (supplemented with >80% DHM). Sisk et al. [39] and Madore et al. [42] compared infants receiving ≥50% MOM to infants receiving ≥50% DHM. The RCT of Schanler et al. [10] randomized infants to receive either DHM or PF as supplementation when insufficient MOM was available, but also provided data on infants who only received MOM. We treated it as observational for this comparison, and compared infants who received exclusive MOM to infants who received MOM supplemented with DHM. 

**Figure 7:**
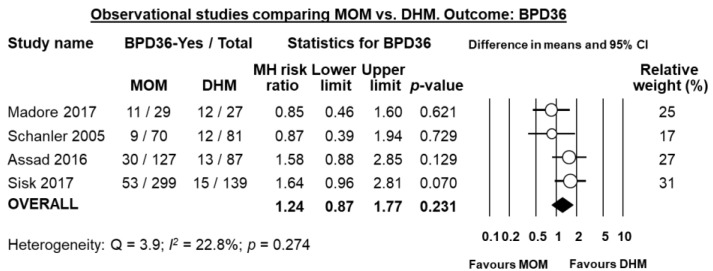
Meta-analysis of observational studies assessing the effects of receiving mainly MOM vs. receiving mainly DHM, on risk of BPD36. Circles (O) represent the effect sizes per study, and their size represents the relative weight of the study in the meta-analysis. Diamonds (◆) represent the pooled effect size. MOM: mother’s own milk; DHM: donor human milk; BPD36: bronchopulmonary dysplasia defined as oxygen dependency at 36 weeks post-menstrual age; MH: Mantel-Haenszel.

Four of the five studies reported on BPD36, and meta-analysis could not find a significant difference in BPD36 risk in the MOM-group compared to the DHM-group (RR 1.24, 95% CI 0.87 to 1.77, *p* = 0.231, Figure 7). The study of Giuliani et al. [34] reported on BPD28 and did not find any significant effect of MOM on this outcome (RR 0.91, 95% CI 0.43 to 1.93, *p* = 0.804). 

### 3.5. Raw Mother’s Own Milk vs. Pasteurized Mother’s Own Milk 

**Figure 8:**
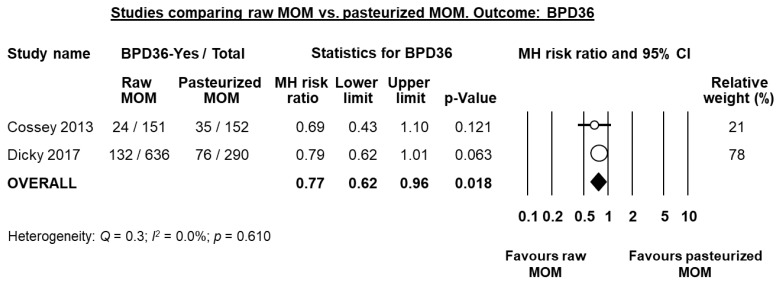
Meta-analysis of studies assessing the effects of receiving raw MOM vs. receiving pasteurized MOM, on risk of BPD36. Circles (O) represent the effect sizes per study, and their size represents the relative weight of the study in the meta-analysis. Diamonds (◆) represent the pooled effect size. MOM: mother’s own milk; BPD36: bronchopulmonary dysplasia defined as oxygen dependency at 36 weeks post-menstrual age; MH: Mantel-Haenszel.

Two studies compared raw or fresh MOM to pasteurized MOM [31,32]. Cossey et al. [31] carried out a RCT where infants were randomized to receive either raw or pasteurized MOM. Dicky et al. [32] carried out a multi-centre observational trial comparing infants of centres where MOM was pasteurized and centres where it was not pasteurized. Individually, either study did not find a significant reduction in BPD36 risk in the raw MOM group when using unadjusted data (Figure 8), although Dicky et al. found a significant reduction on BPD36 risk in the raw MOM group when they adjusted their data for confounders (adjusted OR 0.40, 95% CI 0.27 to 0.67, *p* < 0.001). Since the study of Dicky et al. had a quasi-randomized design, we combined it with the RCT of Cossey et al. in a random effects meta-analysis. This analysis showed a significant reduction of BPD36 risk in the raw MOM group (RR 0.77, 95% CI 0.62 to 0.96, *p* = 0.018, Figure 8).

### 3.6. Raw Mother’s Own Milk vs. Frozen Mother’s Own Milk

One RCT [38] studied the effect of freezing MOM vs. providing it fresh to infants. Infants were randomized to receive only freeze-thawed MOM (frozen at −20 °C for at least three days), or to receive fresh MOM or MOM refrigerated at 4 °C, supplemented with frozen MOM when no fresh MOM was available. The mothers of all included infants had intention to breastfeed. Supplementation in both groups was with pasteurized DHM when no MOM was available. The authors found an increase in BPD36 risk in the group receiving fresh MOM, although it was not significant (RR 1.47, 95% CI 0.98 to 2.21, *p* = 0.062). 

## 4. Discussion

The present study is the first systematic analysis of evidence to date regarding the possible benefits of DHM on BPD. Meta-analysis of RCTs (three studies) could not demonstrate that supplementation of MOM with DHM had a significant effect on BPD36 risk, when compared to supplementation with PF. Meta-analysis of RCTs did find that supplementation with DHM significantly reduced the mean days on mechanical ventilation. Moreover, meta-analysis of observational studies (eight studies) showed a protective effect of DHM supplementation on BPD36 and on days on mechanical ventilation, but not on days on oxygen. Additionally, an exclusive human milk diet (i.e., MOM and/or DHM and DHM-derived fortifier) significantly reduced the risk of BPD36, when compared to a diet that included PF and/or bovine milk-based fortifier. We also found that feeding infants raw MOM, compared to feeding them pasteurized MOM, protected against BPD36. In conclusion, our data suggest that DHM could protect against BPD in very preterm/VLBW infants. However, the process of pasteurization appears to reduce the beneficial properties of human milk on BPD development. 

Our study has several limitations. First, the number of included studies is small. Second, no RCT was primarily designed and powered to detect the effects of DHM on BPD. Third, most RCTs comparing the effects of DHM and PF have included infants receiving some or primarily MOM in both groups because of the inability to ethically assign feeding type [45]. The proportion of MOM and supplementation with either DHM or PF varied across studies. Fourth, there was substantial heterogeneity among the studies in population, timing of initiation, and duration of the intervention. Fifth, the definitions of days on mechanical ventilation and days on oxygen were heterogenous and frequently unclear, and the data itself were not always normally distributed. An additional limitation, inherent to any meta-analysis on BPD, is the heterogeneity of the definition of the condition [46,47,48,49]. Finally, many studies were observational, which potentially reduced the reliability of the results. Despite all these limitations, our meta-analysis shows an additional benefit of using DHM instead of PF in VLBW infants. 

Out of 18 studies we included in our analysis, 12 were published in 2016 or 2017. The use of DHM in the NICU is a topic of much discussion, and the recency of most studies we included reflects this. As more centres start using DHM in clinical practice, and as more RCTs and observational studies are reported, the results of our meta-analyses could change or be confirmed further.

There are several hypothetical mechanisms by which human milk may exert a protective effect against BPD: (i) by improving the nutritional status and growth of the infants; (ii) by reducing postnatal inflammatory processes, such as NEC and LOS; (iii) by modulating the immune functions; and (iv) through the antioxidant properties of human milk. However, the term human milk feeding is frequently used to encompass both MOM and DHM, implying that the multiple beneficial outcomes attributed to MOM can be generalized to DHM [14,15]. This assumption may not be correct because of the important differences between DHM and MOM. As discussed below, part of these differences may be attributed to pasteurization, but there are additional factors that could play a role in the development of outcomes, such as BPD. DHM is typically donated by women who have delivered a term infant and this milk has different levels of nutrients and protective components, including cytokines, growth factors, and lactoferrin, than the milk provided by a mother for her own preterm infant [14,15]. Moreover, it has been suggested that the beneficial effects of MOM may be unique to the specific mother-infant dyad, thereby providing maximum protection to the mother’s own infant [14,15].

The concern about transmission of infectious agents through breast milk led to the obligatory requirement for pasteurization of DHM. Holder pasteurization (62.5 °C for 30 min) of DHM is performed in order to inactivate the microbial agents that may be present [50]. However, pasteurization also destroys or significantly decreases many of the protective elements in human milk, including, among others, lysozymes, secretory immunoglobulin A, growth factors, lactoferrin, antioxidants, and commensal bacteria [14,15,50,51,52]. Although numerous studies have characterized the effects of Holder pasteurization on the biological properties of DHM (see [50] for review), the impact of pasteurization on clinical outcomes has been scarcely investigated. Since DHM should always be pasteurized, it is ethically impossible to study the clinical impact of unpasteurized DHM. However, there are studies comparing pasteurized to unpasteurized MOM. The main purpose of this pasteurization of MOM is the prevention of cytomegalovirus (CMV) infection [53]. 

We included two studies in our systematic review that reported the clinical outcomes of VLBW infants receiving unpasteurized or pasteurized MOM. The study of Cossey et al. [31] was a single-centre RCT, whereas the study of Dicky et al. [32] compared infants from 33 French NICUs that used pasteurized MOM to infants from 30 French NICUs that did not pasteurize MOM. Due to the quasi-randomized design of the study of Dicky et al., we combined it with the study of Cossey et al. in a meta-analysis, which showed that infants fed pasteurized MOM had a significantly higher risk of BPD than infants fed raw MOM (see Figure 8). Therefore, our data suggest that the possible protective effect of human milk on BPD decreases with pasteurization.

As mentioned above, Holder pasteurization not only inactivates all common pathogens in human milk but also affects the commensal microbiota [50]. The microbiota of breast milk has been associated with many of the benefits of raw MOM [54], namely with respect to the prevention of NEC [55,56]. The beneficial effects on the prevention of NEC could not be repeated with pasteurized DHM [30], which may be due to the loss of the microbiota [56,57]. The microbiota of breast milk can be “regrown” in pasteurized DHM with the inoculation of unprocessed MOM [58] which awaits clinical testing. BPD has also been associated with changes in the microbiota of the airways [59,60] and may even be associated with the gut microbiota [61]. Unfortunately, we have no complete understanding about the origin, dynamics and biological function of the respiratory microbiome [62]. Nevertheless, the effect of MOM’s microbiota on the infant gut microbiota and possibly the infant respiratory microbiota may explain our finding on the loss of beneficial effects after pasteurization of human milk. 

In some NICUs, MOM is routinely frozen to reduce the risk of CMV transmission [53]. In our systematic review we included one RCT [38] which studied the effect of freezing MOM compared to providing fresh MOM. They found a close to significant increased risk of BPD in the group receiving fresh MOM (RR 1.47, 95% CI 0.98 to 2.21, *p* = 0.062). It is worth noting that this study was not powered to detect differences in BPD. Further studies are needed to elucidate the effect of MOM-freezing on neonatal outcomes.

Both MOM and DHM must be fortified to provide sufficient support for growth and development in the postnatal period of the VLBW infant [63,64,65,66]. At present, most NICUs use bovine milk-derived fortifiers. However, the recent availability of a DHM-derived fortifier has led to the introduction of the concept of “exclusive human milk diet” [28,29,35,40]. This diet consists of MOM, DHM if MOM is not adequately available, and fortification with DHM-derived fortifier. Our data suggest that this exclusive human milk diet may reduce the risk of developing BPD. However, this result should be interpreted with care. Most studies evaluating the use of an exclusive human milk diet [29,35,40] do not isolate the individual effects of the DHM-derived fortifier, because the group receiving bovine products is a mixture of infants fed DHM plus bovine milk-derived fortifier and infants fed PF. Therefore, it is not possible to determine whether the lower risk of BPD was due to the benefit of DHM-derived fortifier or to the benefit of avoiding PF. Only the study of Assad et al. [28] provided data on infants not exposed to PF and could not find a significant change in BPD risk when using DHM-derived fortifier, compared to bovine-derived fortifier. 

Maintaining a sufficient (unpasteurized) MOM supply is challenging for mothers of VLBW infants, necessitating supplementation with either DHM or PF. Our results suggest the superiority of DHM over PF in reducing BPD, but we should not forget that MOM is more effective in the reduction of multiple morbidities and is less expensive to acquire than DHM. Therefore, NICU care providers should prioritize interventions to support initiation and maintenance of lactation and make efforts to identify and solve lactation problems in mothers of VLBW infants.

## Figures and Tables

**Table 1:** Synoptic table of characteristics of included randomized controlled trials.

Authors	*n* of Infants (Centers)	Inclusion Criteria	Primary Outcome(s)	Respiratory Outcome(s)	Studied Intervention	Duration of Intervention	Fortification	Comments
Corpeleijn et al. 2016 [30]	373 (6)	BW <1500 g, MOM insufficiently available.	Composite incidence of NEC, serious infection (sepsis or meningitis), or all-cause mortality between 72 h and 60 days of life.	BPD28	- MOM + DHM - MOM + PF	10 days or hospital transfer or death.	Bovine fortifier added to MOM and DHM.	Median MOM intakes were higher in the DHM group, 89.1% in the DHM group vs. 84.5% in the PF group.
Cossey et al. 2013 [31]	303 (1)	GA <32 weeks, BW <1500 g.	Incidence of proven late-onset sepsis.	BPD36, days on MV	- Raw MOM + PF - Pasteurized MOM + PF	Eight weeks or discharge.	Not specified.	MV defined as respiratory support.
Cristofalo et al. 2013 [43]	53 (7)	BW 500–1250 g, no intention to provide MOM, parenteral nutrition within 48 h, enteral feeding within 21 days.	Duration of parenteral nutrition.	Days on MV, days on O_2_	- Exclusive DHM - Exclusive PF	91 days of age or discharge, or attainment of 50% oral feedings.	DHM fortifier added to DHM.	
O’Connor et al. 2016 [41]	363 (4)	BW < 1500 g, enteral feeding within 7 days.	Bayley-III score at 18 months.	BPD36	- MOM + DHM - MOM + PF	90 days or discharge.	Bovine fortifier added to MOM and DHM.	MOM + DHM group: MOM = 58% of intake. MOM + PF group: MOM = 63% of intake.
Omarsdottir et al. 2015 [38]	140 (2)	GA < 28 weeks, intention to provide MOM, intention to breastfeed.	CMV transmission to infants from breast milk, rate of symptomatic CMV infection.	BPD36	- Frozen MOM + DHM - Fresh MOM + frozen MOM + DHM	Until 32 weeks PMA.	Not specified.	Frozen MOM: stored for ≥3 days at −20 °C. Fresh MOM: fed immediately or after refrigeration at +4 °C.
Schanler et al. 2005 [10]	243 (1)	GA ≤ 29 weeks.	Incidence of late-onset sepsis and/or NEC.	BPD36, days on MV	- MOM + DHM - MOM + PF	90 days or discharge.	Bovine fortifier added to MOM and DHM.	
Sullivan et al. 2010 [40]	207 (12)	BW 500–1250 g, intention to provide MOM.	NEC.	BPD36, days on MV, days on O_2_	- MOM + DHM + DHM fortifier - MOM + PF + bovine fortifier	91 days or ≥50% oral feedings or discharge.	Donor DHM-based fortifier in DHM group, bovine fortifier in PF group.	

BPD28: bronchopulmonary dysplasia, defined as oxygen dependency at day 28 of life; BPD36: bronchopulmonary dysplasia, defined as oxygen dependency at 36 weeks corrected gestational age; MOM: mother’s own milk; PF: preterm formula; DHM: donor human milk; MV: mechanical ventilation; GA: gestational age; BW: birth weight; PMA: post-menstrual age; CMV: Cytomegalovirus; NEC: necrotizing enterocolitis.

**Table 2:** Synoptic table of characteristics of included observational studies.

Authors	Study Design	*n* of Infants (Centers)	Inclusion Criteria	Primary Outcome(s)	Respiratory Outcome(s)	Groups	Duration of Intervention	Fortification	Comments
Assad et al. 2015 [28]	Retrospective cohort	293 (1)	GA < 29 and/or BW ≤ 1500 g.	Feeding intolerance, time to full feeds, length of stay.	BPD36	- MOM + DHM + DHM-based fortifier; - MOM + bovine fortifier; - MOM + PF + bovine fortifier; - Exclusive PF	Until discharge.	DHM fortifier in exclusive human diet, bovine fortifier in bovine groups.	
Colacci et al. 2017 [29]	Retrospective cohort	85 (1)	BW < 1000 g, GA < 37 weeks.	Neurodevelopmental impairment (Bayley-III score).	BPD36	- MOM + DHM + DHM-based fortifier; - MOM + PF + bovine fortifier	At least four weeks, until weight ≥ 1500 g, or 34 weeks PMA.	DHM-based fortifier in exclusive human milk group, bovine fortifier in other group.	
Dicky et al. 2017 [32]	Retrospective cohort	926 (63)	GA < 32 weeks, alive at 7 days of life.	In-hospital mortality, short-term morbidity, weight gain.	BPD36, Days on MV, Days on oxygen	- Raw MOM + PF/DHM; - Pasteurized MOM + PF/DHM	Until 33 weeks corrected age or until discharge.	Bovine fortifier added to both groups.	The supplement (PF, DHM, infant formula) to raw or pasteurized MOM varied per center.
Ginovart et al. 2016 [33]	Retrospective cohort	186 (1)	BW < 1500 g.	Retinopathy of prematurity.	BPD28, BPD36, days on MV, days on O_2_	- MOM + DHM; - MOM + PF	Four weeks.	Bovine fortifier added to MOM and to DHM.	Additional data provided by authors, and from later (2017) report of Ginovart et al. [ 44].
Giuliani et al. 2012 [34]	Retrospective cohort	92 (1)	GA > 23 weeks, BW < 1500 g.	Growth, and short-term clinical outcomes.	BPD28	- Mainly MOM (>80%); - Mainly DHM (>80%)	32 weeks corrected GA.	Bovine fortifier added to MOM.	
Hair et al. 2016 [35]	Retrospective cohort	1587 (4)	BW < 1250 g.	NEC, mortality.	BPD36, days on MV	- MOM + DHM + DHM-based fortifier; - MOM + PF + bovine fortifier	32–34 corrected GA, or 60 days of life. depending on the centre.	DHM-based fortifier in DHM group. Bovine fortifier in bovine group.	
Kreissl et al. 2017 [36]	Prospective cohort	283 (1)	GA < 32 weeks, BW < 1500 g.	Time to full enteral feeding.	BPD36	- MOM + DHM; - MOM + PF	Until term or discharge. DHM group received DHM until MOM was available or until reaching 140 mL/kg/day, then switched to term formula.	Bovine fortifier added to MOM and DHM when infant reached intake of 100 mL/kg/day.	DHM provided by other preterm mothers.
Lee et al. 2016 [37]	Retrospective cohort	46 (1)	BW < 1500 g.	Morbidity, duration parenteral nutrition, length of hospital stay.	BPD36, days on MV, days on O_2_	- MOM + DHM; - MOM + PF	Not specified.	Not specified.	Days on MV: defined as invasive ventilation. Days on O_2_ defined as non-invasive ventilation.
Madore et al. 2017 [42]	Retrospective case-control	81 (1)	BW < 1000 g.	Growth, neurodevelopment.	BPD36	- Exclusive MOM; - DHM > 50%; - PF > 50%	First month of life.	Bovine fortifier added to MOM and to DHM.	
Sisk et al. 2017 [39]	Retrospective cohort	563 (1)	GA ≤ 32 weeks and BW ≤ 1500 g.	NEC stage ≥ 2.	BPD36	- MOM ≥ 50%; - DHM ≥ 50%; - PF ≥ 50%	Within 2 h of birth until 34 weeks PMA.	Bovine fortifier added to MOM and to DHM.	
Spiegler et al. 2016 [21]	Prospective cohort	1433 (48)	GA 22–31 6/7, BW < 1500 g.	BPD.	BPD36, days on MV, days on O_2_	- MOM + DHM; - MOM + DHM + PF; - Exclusive PF	Until discharge.	Bovine fortifier added to MOM and to DHM.	

BPD: bronchopulmonary dysplasia; BPD28: bronchopulmonary dysplasia defined as oxygen dependency after day 28 of life. BPD36: bronchopulmonary dysplasia, defined as oxygen dependency at 36 weeks corrected gestational age; MV: mechanical ventilation; MOM: mother’s own milk; PF: preterm formula; DHM: donor human milk; GA: gestational age; BW: birth weight. PMA: post-menstrual age. NEC: necrotizing enterocolitis.

## Supplementary Materials

The following are available online at http://www.mdpi.com/2072-6643/10/2/238/s1, Table S1: PRISMA 2009 checklist.

## Appendix A

**Table A1:** Assessment of risk of bias of included randomized controlled trials.

Study	Random Sequence Generation	Allocation Concealment	Blinding of Participants and Personnel	Blinding of Outcome Assessment	Incomplete Outcome Data	Selective Reporting	Other Bias
Corpeleijn et al. 2016 [30]	LR	LR	LR	LR	LR	LR	LR
Cossey et al. 2013 [31]	LR	LR	HR	UR	LR	LR	LR
Cristofalo et al. 2013 [43]	LR	LR	LR	LR	LR	LR	LR
O’Connor et al. 2016 [41]	LR	LR	LR	LR	LR	LR	LR
Omarsdottir et al. 2015 [38]	UR	UR	HR	UR	LR	LR	LR
Schanler et al. 2005 [10]	LR	LR	HR	HR	LR	LR	LR
Sullivan et al. 2010 [40]	LR	LR	HR	LR	LR	LR	LR

LR: low risk of bias, HR: high risk of bias, UR: unknown risk of bias.

**Table A2:** Assessment of methodological quality of included observational studies, using the Newcastle-Ottawa Scale.

Study Name	Study Design	Selection (0–4 Points)	Comparability (0–2)	Outcome (0–3)	Total (0–9)
Assad et al. 2015 [28]	Retrospective cohort	4	0	3	7
Colacci et al. 2017 [29]	Retrospective cohort	4	0	3	7
Dicky et al. 2017 [32]	Retrospective cohort	4	2	3	9
Ginovart et al. 2016 [33]	Retrospective cohort	4	0	3	7
Giuliani et al. 2012 [34]	Retrospective case-control	4	0	3	7
Hair et al. 2016 [35]	Retrospective cohort	4	0	3	7
Kreissl et al. 2017 [36]	Prospective cohort	4	0	3	7
Lee et al. 2016 [37]	Retrospective cohort	4	0	2	6
Madore et al. 2017 [42]	Retrospective case-control	4	2	3	9
Sisk et al. 2017 [39]	Retrospective cohort	4	0	3	7
Spiegler et al. 2016 [21]	Prospective cohort	4	2	3	9
